# Preliminary Support for the Use of Motivational Interviewing to Improve Parent/Adult Caregiver Behavior for Obesity and Cancer Prevention

**DOI:** 10.3390/ijerph20064726

**Published:** 2023-03-07

**Authors:** Ashlea Braun, James Portner, Menglin Xu, Lindy Weaver, Keeley Pratt, Amy Darragh, Colleen K. Spees

**Affiliations:** 1 Department of Nutritional Sciences, College of Education and Human Sciences, Oklahoma State University, Stillwater, OK 74078, USA; 2 College of Social Work, The Ohio State University, Columbus, OH 43210, USA; 3 The Ohio State University Wexner Medical Center, Columbus, OH 43210, USA; 4 Division of Occupational Therapy, School of Health and Rehabilitation Sciences, The Ohio State University College of Medicine, Columbus, OH 43210, USA; 5 Human Development and Family Science Program, Department of Human Sciences, College of Education and Human Ecology, The Ohio State University, Columbus, OH 43210, USA; 6 Department of Surgery, The Ohio State University Wexner Medical Center, Columbus, OH 43210, USA; 7 Department of Pediatrics, The Ohio State University College of Medicine, Columbus, OH 43210, USA; 8 Division of Medical Dietetics, School of Health and Rehabilitation Sciences, The Ohio State University College of Medicine, Columbus, OH 43210, USA; 9 Arthur G. James Cancer Hospital and Richard J. Solove Research Institute, The Ohio State University Wexner Medical Center, Columbus, OH 43210, USA

**Keywords:** motivational interviewing, obesity, obesity prevention, nutrition therapy, health behavior, diet

## Abstract

Motivational interviewing (MI) is a promising behavioral intervention for improving parent and adult caregiver (PAC) health behavior for obesity and cancer prevention. This study explored the preliminary effects of MI from a registered dietitian (RDMI) within an obesity prevention intervention to promote PAC behavior change and positive proxy effects on children and the home environment. N = 36 PAC/child dyads from low-resource communities were enrolled in a randomized trial testing a 10-week obesity prevention intervention. Intervention dyads were offered RDMI sessions. Data were collected at baseline and post-intervention (PAC diet quality (Healthy Eating Index (HEI)), child skin carotenoids, home environment, and PAC ambivalence regarding improving diet). Results show that for every RDMI dose, PAC HEI scores increased (0.571 points, *p* = 0.530), child skin carotenoid scores improved (1.315%, *p* = 0.592), and the home food environment improved (3.559%, *p* = 0.026). There was a significant positive relationship between RDMI dose and change in ambivalence (ρ = 0.533, *p* = 0.007). Higher baseline ambivalence was associated with greater dose (ρ = −0.287, *p* = 0.173). Thus, RDMI for PACs may improve diets among PACs who are otherwise ambivalent, with potential effects on the diets of their children and the home food environment. Such intervention strategies have the potential for greater effect, strengthening behavioral interventions targeting obesity and cancer.

## 1. Introduction

The presence of obesity has been identified as a key factor in the development of multiple preventable cancer types [1,2,3,4]. Sufficient consumption of fruits and vegetables and overall dietary patterns contribute to decreased risk for certain cancers, as well as obesity, which further mitigates cancer risk [2]. In the United States (US), rates of obesity among adult and youth populations alike have been steadily increasing, while fruit and vegetable consumption remains suboptimal [5,6,7,8]. Given the challenges associated with treating obesity in adulthood as a means of cancer prevention and control, obesity prevention in youth is essential [9]. Critical to effective obesity prevention are multidimensional strategies, including family-based prevention efforts, to simultaneously address parents and adult caregivers’ (PACs) and children’s health behavior (e.g., diet) [10,11,12,13,14]. Motivational interviewing (MI) has an established empirical base for obesity prevention efforts that include PACs [15]. Previous MI-based interventions have targeted PACs to improve adherence in child-based interventions [16], encourage weight loss in children [17], change child behavior or family-related factors (e.g., family meals) [18,19,20,21,22,23], and have been delivered in groups to families [24,25,26,27,28] or directly to children in the presence of PACs [16,29,30,31,32,33,34]. Many of these studies suffer from design limitations, as they do not specifically or adequately define, and subsequently target, PAC behaviors as a catalyst for youth behavior change and obesity prevention [10,35,36,37,38]. Given PACs can influence child behavior (e.g., modeling), there is potential for PAC-delivered interventions to indirectly influence child behavior, versus directive approaches that may be frustrating for families if child compliance is poor [39,40,41,42].

Given the novelty of PAC-focused MI, rigorous methods are warranted (e.g., use of trained interventionists, fidelity monitoring, integration with behavior- or outcome-specific treatment modalities) [18,43,44,45,46,47,48,49,50]. The efficacy of MI is strengthened when combined with behavior- and/or outcome-specific treatments [45,51]. As such, the use of registered dietitians (RDs) is a logical extension of MI targeting dietary patterns [18,44,52]. Integration is challenging, and a thorough understanding of implementation is critical [53]. The purpose of this study was to explore the preliminary efficacy of PAC behavior-focused MI from an RD (RDMI), in order to improve diet- and health-related outcomes and the home/family environment, among dyads enrolled in an obesity prevention intervention. A secondary purpose was to characterize implementation. The authors hypothesized that RDMI would contribute to an improved diet in the PACs and children, and that greater improvements would be seen with greater doses.

## 2. Materials and Methods

### 2.1. Participants and Recruitment

Parents/adult caregivers and one 8–9-year-old child from the central Ohio area were invited to participate as dyads. Recruitment occurred via communication with local schools that received federal funding for free, as well as reduced-price, breakfast and lunch to students, in order to target dyads from low-resource/under-resourced communities. Interested PACs were screened for eligibility prior to scheduling a baseline orientation/data collection visit. Additional inclusion criteria included the following: (1) fluent English-speaking dyads and (2) ability to consume fruits and vegetables without concerns of medication–nutrient interactions. Exclusion criteria included the following: (1) diagnosed with mental, physical, or communication disabilities, or difficulties that would impair full participation in all components of the intervention; (2) lack of transportation to weekly classes; (3) non-English speaking; (4) consuming over-the-counter herbals, botanicals, or nutritional supplements (excluding multivitamins); (5) diagnosed with active metabolic or digestive illnesses, which may result in nutrient malabsorption. Participating caregivers were required to be a primary caregiver responsible for food procurement and preparation for the participating child, though no specific relationship was required (e.g., mother/father, aunt/uncle). All procedures were performed in accordance with the ethical standards of The Ohio State University’s institutional review board and the 1964 Declaration of Helsinki and its later amendments or comparable ethical standards.

### 2.2. Study Design

**Figure 1:**
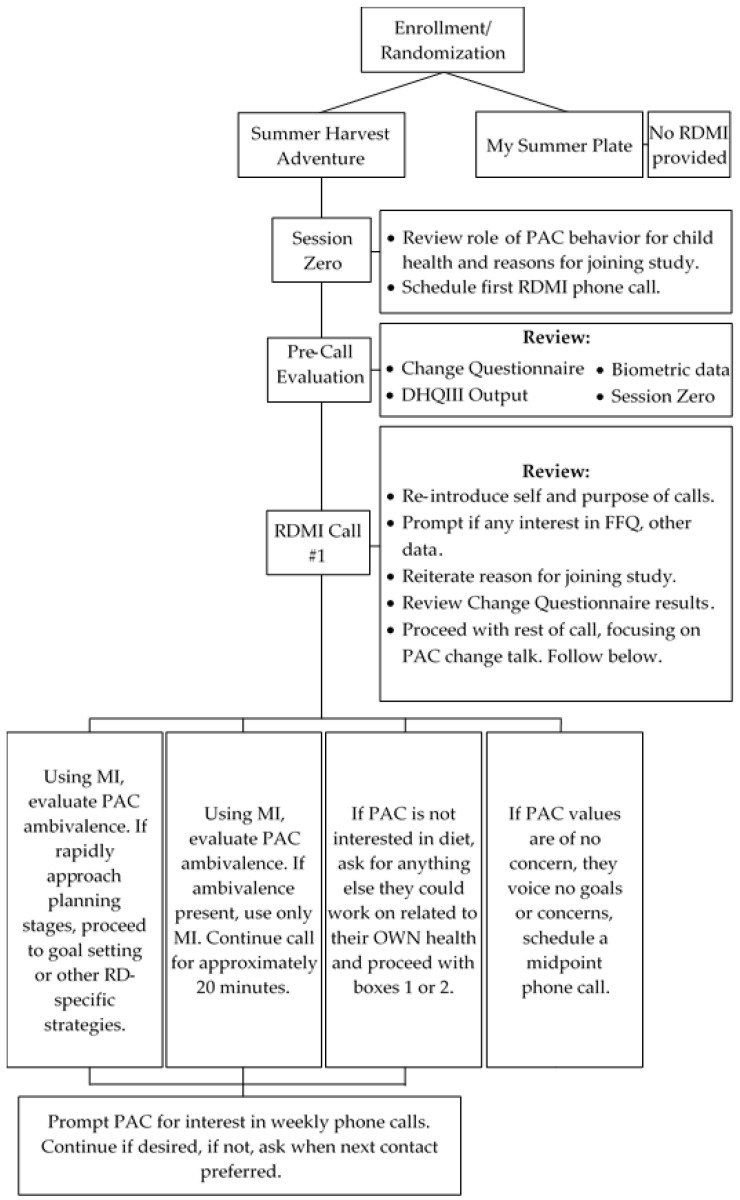
Overall Study Design and Protocol for RDMI.

The Summer Harvest Adventure is a five-year, randomized controlled trial designed to determine the efficacy of a multicomponent obesity prevention intervention to improve the consumption of fruits and vegetables in youth; RDMI was offered as one intervention component. Enrolled participants were randomized to the intervention, The Summer Harvest Adventure, or an education-only control, My Summer Plate (Figure 1). For the study presented herein, in-depth data were collected among only those dyads randomized to the Summer Harvest Adventure in year 2, for an evaluation of methods and processes. The ten-week intervention included weekly (1) group child-focused education; (2) garden harvesting of fruits, vegetables, and herbs; and (3) RDMI for PACs. In total, descriptions of the study are informed by the Template for Intervention Description and Replication (TIDieR) checklist [54]. The Summer Harvest Adventure intervention has been described elsewhere (Clinicaltrials.gov: NCT05367674) [55,56].

### 2.3. Weekly Intervention Sessions

Each week of the intervention included education focused on obesity-preventive behaviors, including foundational healthy habits (e.g., avoidance of sugar-sweetened beverages), with an emphasis on fun physical activity and improving adherence to the US Dietary Guidelines for Americans [57,58]. Sessions included cooking demonstrations and taste tests, interactive lessons, and kinetic activities to integrate “play” into the week’s lesson. After each session, dyads could harvest produce from a professionally maintained 2.5-acre urban garden.

### 2.4. Remote Motivational Interviewing from a Registered Dietitian (RDMI)

As part of the intervention, each PAC was introduced to the RD providing RDMI at baseline data collection visits (Figure 1). Here, participants were explained the purpose/structure of RDMI, asked for reasons for study enrollment, and asked to schedule an initial phone call for the first week of the ten-week intervention [59]. The purpose of RDMI was framed as being focused on the PAC’s behavior related to improving their own diet quality, not the child’s, but that such change could have a positive impact on the child’s behavior. Thereafter, the structure of this phone call occurred using methodology integrating MI with MNT, nutrition counseling, or nutrition education, as well as more directive styles consistent with Self-Determination Theory [60]. Initial phone calls lasted approximately 20 min, though could be altered as needed [61]. After completion of this initial call, contact was planned collaboratively based on the RD’s evaluation and PAC-communicated needs/preferences. In the instance of an unsuccessful call, the RD would attempt again the following week. Contact ceased after three unsuccessful interactions (e.g., no response, no return call) [62].

RDMI interactions focused on issues related to the dietary patterns of PACs; PACs were able to discuss other related topics of interest (e.g., weight loss) if desired. If PACs brought up questions regarding their child’s health (e.g., picky eating), the RD would answer these questions by drawing connections to PAC’s own dietary patterns. If requested, additional resources were delivered to PACs via email, however, counseling occurred only via phone.

### 2.5. Data Collection

Comprehensive data collection visits occurred before and after (i.e., baseline and post-intervention) the ten-week intervention period for dyads, during which data were recorded using Research Electronic Data Capture (REDCap) tools hosted at The Ohio State University [63]. Additionally, data regarding PAC RDMI interactions were recorded, including the dates of interactions, summaries of PAC’s responses, and lengths of phone calls.

### 2.6. Survey Measurements

At baseline, sociodemographic questions adapted from the Behavioral Risk Factor Surveillance System were administered [64]. Parental modeling was assessed via the Diet Structure Scale within the validated Home Self-Administered Tool for Environmental Assessment of Activity and Diet Family Food Practices Survey (HomeSTEAD) [65]. Items included six Likert-scale questions, with responses ranging from 1–5, e.g., “My child learns to eat healthy snacks from me.” An average score across items was computed; higher scores indicate more PAC modeling.

Attitudinal ambivalence was assessed using the validated Change Questionnaire [66,67]. This includes 12 questions related to a specified target behavior, which for the present study was “improve the foods that you eat.” Each question incorporates a different aspect of “change talk” (e.g., “I want to make this change,” “I have to make this change,”), and is rated on a scale from 0 (“definitely not”) to 10 (“definitely”); higher scores indicate less ambivalence.

### 2.7. Dietary Patterns and Diet Quality

PACs completed the National Cancer Institute’s Diet History Questionnaire III (DHQIII) [68]. Upon completion, DHQIII computes Healthy Eating Index 2015 (HEI) scores in order to assess overall diet quality, based upon adherence to the 2015–2020 US Dietary Guidelines [57,69]. Total HEI scores range from 0 to 100; a score of 100 represents maximal adherence. Children’s skin carotenoids were measured in triplicate on the palm of their right hand via resonance Raman spectroscopy using the Pharmanex NuSkin Biophotonic Scanner S3 (NuSkin Enterprises, Provo, UT, USA). Means were computed for a final score, reported in Raman intensity counts (RSS counts).

### 2.8. Anthropometric and Clinical Measurements

For dyads, height was measured with a seca 213 portable stadiometer (seca North America, China, CA). Weight was measured on a Tanita SC-331S Total Body Composition Analyzer (Tanita, Arlington Heights, IL, USA). Height and weight were used to calculate body mass index (BMI) for PACs in kg/m^2^, as well as the BMI percentile for children, based upon age and sex.

### 2.9. Interaction and Process Data

Data on all interactions with the RD were recorded. Each interaction was coded as a reciprocal interaction (RI) if the participant responded to the RD’s communication in any capacity, regardless of the content of the response. Each RI was then further classified as a dose of RDMI if the RD and participant engaged in a two-way interaction that resulted in MI, MNT, nutrition counseling, or nutrition education. Based on this coding, use was defined four ways: (1) *RI Dose Received* (RI), or a summation of the total number of individual contacts from the RD to which the participant responded at all, regardless of content of that response; (2) *RDMI Dose Received,* or the number of individual RIs that included the PAC’s thorough response to the delivery of MI, MNT, nutrition counseling, or nutrition education; (3) *RDMI Time Received*, or the total time (in minutes) of interaction with the RD that resulted in RDMI; 4) *RDMI Engagement*, calculated by dividing the number of completed RDMI interactions by the number of attempted interactions, computing a total percentage. PACs were classified as *RDMI Completers* or *RDMI Non-Completers* based on whether or not communication ceased after three consecutive failed attempts (i.e., reaching this cut-off point led to classification as a *RDMI Non-Completer*).

The Health Care Climate Questionnaire (HCCQ) was administered to PACs at post-intervention to assess their perceived autonomy [70,71]. The HCCQ includes 15 items, each rating a different aspect of provider care (e.g., “I feel that my [provider] has provided me choices and options”). Each item is rated from 1–7, with an average of all items computed; higher scores indicate a greater emphasis on autonomy. Lastly, included in programmatic evaluations were questions related to satisfaction with the RDMI, which were administered at post-intervention, including “How would you rate the health coaching with [the dietitian]?” (rated on a five-point scale from poor to excellent).

### 2.10. Attendance and Participation

Dyad participation in the other intervention components was collected. At each weekly session, dyad attendance was collected. Although dyads were encouraged to attend these activities together, PACs could attend independently if the child was unable to do so, while children could attend alone as long as they were accompanied by an adult caregiver. Ten total sessions were offered, and the total number of sessions attended by either member of the dyad were summated.

### 2.11. Fidelity to MI

With PAC approval, phone calls were audio-recorded for the purposes of MI fidelity assessment. Recordings were selected at random and reviewed with the Motivational Interviewing Treatment Integrity Tool (Version 4.2.1) [47]. A total of 10% of all phone calls were randomly selected and reviewed for fidelity, and all met the benchmark for “good” MI, with the exception of the reflections to questions ratio, which reached an average of 1.6:1 [45,47].

### 2.12. Statistical Analyses

Descriptive statistics were computed for PAC socio-demographics, baseline characteristics, and measures of RDMI use. For the purposes of baseline descriptions, the cohort was defined in three ways: (1) all PACs who were classified as *RDMI Completers*; (2) all PACs randomized to the Summer Harvest Adventure who completed baseline and post-intervention assessments, regardless of RDMI use; (3) all PACs randomized to the Summer Harvest Adventure at enrollment regardless of follow up. Logistic regression was used to explore the relationship between PAC race (African American or white); baseline PAC BMI, PAC HEI, and Change scores; baseline child BMI percentile; and classification as a *RDMI Completer*. These variables were selected based on influences considered relevant based on the theoretical basis of the intervention (i.e., ambivalence), as well as potential influences, per the existing literature [72,73,74]. To explore the relationship between measures of RDMI use and both baseline measures of and changes in ambivalence, Spearman correlations were conducted given non-normality of data.

Pearson correlations were used to explore the relationship between measures of RDMI use, percent change in HEI scores, and percent weight change from baseline to post-intervention; Spearman correlations were used to evaluate the correlation between *RDMI Time Received* and relevant outcomes due to non-normality of data. Lastly, individual multiple linear regressions were conducted to explore the relationships between measures of RDMI use and changes in PAC diet quality, child skin carotenoid scores, and PAC modeling, while controlling for dyad participation in the other components of the Summer Harvest Adventure. Unless otherwise specified, all assumptions were evaluated and confirmed based on visual and statistical inspection of data. Analyses were completed using SPSS (Version 25 or 26) and statistical significance was set at *p* ≤ 0.05.

## 3. Results

### 3.1. Baseline Characteristics and Measures of RDMI Use

**Figure 2:**
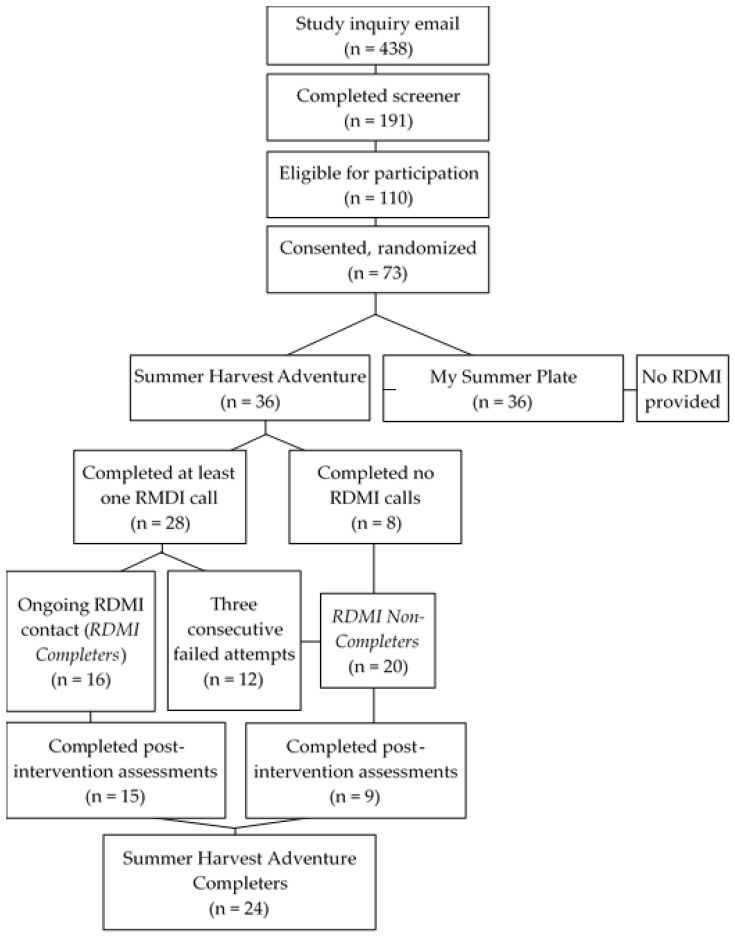
Modified Consort Diagram.

Thirty-six PACs were enrolled in the Summer Harvest Adventure; 78% (n = 28) participated in at least one RDMI session, and 44% (n = 16) were considered *RDMI Completers* (Figure 2). Overall, a large portion of PACs were African American, and the average age of *RDMI Completers* was 38 years (SD = 6), while the majority were female (81%), married (44%), and employed (94%) (Table 1). Measures of RDMI use indicated that among PACs who attended the baseline and post-intervention assessments, an average of three RDMI doses were received, equating to a total of 47.6 minutes of RDMI delivered. Among *RDMI Completers*, *RDMI Engagement* was 70%, and all measures of RDMI use increased sequentially among those randomized to the Summer Harvest Adventure, those being Summer Harvest Adventure completers, and those classified as *RDMI Completers*, respectively (with the exception of *RDMI Dose Received*) (Supplementary Table S1).

Logistic regression was utilized to determine the likelihood of classification as a *RDMI Completer* versus *RDMI Non-Completer* (Supplementary Table S2). The odds of RDMI completion increased with increasing PAC BMI (OR = 1.080), indicating that for every one unit increase in PAC BMI, the odds of being classified as a *RDMI Completer* increased by 8%. Odds of RDMI completion decreased with increasing baseline child BMI percentile (OR = 0.997), baseline PAC HEI (OR = 0.992), baseline Change scores (OR = 0.652), and among those identified as African American (OR = 0.545).

### 3.2. Ambivalence

A strong statistically significant relationship was found between percentage change in Change score from baseline to post-intervention and *RDMI Dose Received* (ρ = 0.533, *p* = 0.007). Overall, baseline Change scores were negatively correlated with all measures of RDMI use, including *RDMI Time Received* (ρ = −0.287, *p* = 0.173) (indicating higher ambivalence was associated with higher measures of dose).

### 3.3. PAC Clinical Outcomes and Diet Quality

A weak positive correlation between measures of RDMI use and percentage weight change among PACs existed (e.g., *r* = 0.291, *p* = 0.178 for *RI Dose Received*). Weak to strong positive correlations existed between measures of RDMI use and percent change in the PAC HEI scores from baseline to post-intervention (*RI Dose Received*, *RDMI Dose Received*, *RDMI Time Received*, *RDMI Engagement* (*r* = 0.250, *p* = 0.238; *r* = 0.342, *p* = 0.102; ρ = 0.477, *p* = 0.018; *r* = 0.358, *p* = 0.086, respectively)). Differing contributions of various representations of RDMI use were observed in predicting the raw change in PAC HEI scores (Table 2). The lowest predictive capacity was seen in *RDMI Time Received* (*B* = 0.055), and was the highest for *RDMI Dose Received* (*B* = 0.980). These data indicate that for every one unit increase in *RDMI Time Received*, a 0.055 point increase was noted in HEI score, versus *RDMI Dose Received*, which, for every one unit increase, a 0.980 increase in HEI score was noted.

### 3.4. Child and Home-Related Outcomes

The lowest contribution towards child skin carotenoids was noted for *RDMI Engagement* (β = −0.047, *B* = −0.027), and the highest was for *RI Dose Received* (β = 0.128, *B* = 1.315) (Table 3). That is, for every one unit increase in *RI Dose Received*, a 1.315% increase in child skin carotenoid scores were noted. Regarding changes to the home environment (Table 4), similar trends were noted: a lower contribution was found for *RDMI Engagement* (*B* = 0.088, β = 0.228) versus *RI Dose Received* (*B* = 3.559, β = 0.506), the latter of which was significant (*p* = 0.026). That is, for every one unit increase in *RI Dose Received*, a 3.559% increase in PAC modeling was observed. Collectively, Summer Harvest Adventure attendance and *RI Dose Received* accounted for the greatest variability in both child skin carotenoids (adjusted R^2^ = −0.055, *f*^2^ = 0.037) and PAC modeling (adjusted R^2^ = 0.144, *f*^2^ = 0.279).

### 3.5. Programmatic Evaluations and Autonomy

Fifty-eight percent of PACs (n = 14) rated RDMI as “excellent”, 21% (n = 5) as “very good”, and 4% (n = 1) as “good”; 17% (n = 4) stated they “did not use” RDMI. Of those, n = 2 did not engage in any RDMI interactions. However, n = 2 did, reporting “Did not really know what to discuss…and find the recipes helpful we learned at the garden study…” and “I am already well-versed in nutrition and healthy living and employ those practices in my everyday life, consistently.” Participants who completed at least one RDMI session reported a mean score of 6.5 out of 7 (SD = 0.87, range 4.3–7) on the Health Care Climate Questionnaire.

## 4. Discussion

The objective of this study was to explore the preliminary effects of PAC-focused RDMI on outcomes among dyads in a multicomponent obesity prevention intervention. Addressing PACs in this context is critical [13]. The current study provides initial support for use of MI as a means to do so, as it facilitates supportive environments for children to learn and modify behavior through social learning, corroborating previous work [29,75,76]. There is a large body of work regarding the application of MI, with a direct emphasis on child behavior [32]; this study fills a gap regarding the utility of MI focused on PAC behavior.

A primary advantage of using MI is its focus on the psychological and/or cognitive predictors of behavior change (e.g., ambivalence) versus an emphasis on passive education/knowledge, or more paternalistic approaches. Such an appreciation for the varying predictors of, and/or contributors to health behavior and its related outcomes (e.g., psychological factors, mental health), is essential for prevention strategies. This characteristic of MI may contribute to its potentially positive effect on health outcomes among underserved populations, including diverse populations [77]. The present cohort consisted, primarily, of women who self-identified as African American. Some previous MI literature has suggested MI may not be as well-received by African American populations [78]. However, the cohort in this study responded well, which may be the result of the integrated approach. African American cohorts may prefer more directive styles of counseling, thus the flexible approach taken in this study may be advantageous [60,73,78,79]. This is reflected in MI fidelity assessments (e.g., a lower reflections-to-questions ratio (1.16:1)).

African American participants were more likely to be classified as *RDMI Non-Completers*, however, this provides evidence for a need to further understand the appropriate tailoring of the design and understanding the ideal dose of RDMI, and does not indicate poor compliance per se. In reality, the majority of participants completed more than one RDMI interaction, with improvements in outcomes (e.g., diet quality), thus the criteria for “completion” may be too exclusionary and require future revisions. An average of three to four doses were delivered, and as few as one dose of MI may modify behavior. Focusing on number of doses, rather than rate of completion of planned doses, may be a more appropriate approach [80,81]. Further examination of race/ethnicity, as well as other demographic variables (e.g., marital status, education), as potential moderators should be conducted in future MI research, and be used to adapt more culturally or socially tailored approaches.

This study documents that RDMI use was associated with improvements in PAC health behavior. Specifically, for every one-unit increase in *RDMI Dose Received*, there was a 0.98-point increase in HEI scores. This equates to an improvement of nearly ten points if one RDMI session was completed every week of the 10-week intervention. Though these results were not significant, this may be secondary to the small sample size, and nonetheless provide an indication of potential impacts that may be elucidated in fully-powered trials. In various populations, HEI improvements greater than five points are associated with positive health outcomes [82,83]. Interestingly, child skin carotenoids did not similarly respond to RDMI, but instead responded to general dose, regardless of content (i.e., *RI Dose Received*). Similar trends were observed for PAC Modeling, suggesting that the quality of interactions was a greater predictor of change within PACs, but for child or family-related outcomes, quantity of interactions was paramount. This suggests differing mechanisms. Although research indicates positive effects on children when PACs engage in health behavior, dieting can negatively impact a child’s health if it results in negative messaging in the home [84,85]. Though the present study focused on PAC diet quality (i.e., not dieting) it is possible that those more likely to engage with the RDMI could be delivering different indirect messages in the home.

Further, RDMI in this study was one component of a multicomponent intervention, and interest remains in understanding the unique contributions of RDMI as an “active ingredient.” Attendance/participation in other study components did contribute to diet-related outcomes, though to varying degrees, and in some instances, RDMI demonstrated a greater contribution. *RDMI Engagement* and Summer Harvest Adventure attendance/participation accounted for the greatest variability in PAC diet quality (i.e., 9.5%). Regarding changes in child skin carotenoids, RDMI had a substantial impact on the context of the intervention, which may provide support for options enabling remote intervention delivery, to account for families’ busy schedules and to address common issues (e.g., travel) [86,87,88].

Results of logistic regression indicate that PAC BMI was the strongest predictor of RDMI completion. The literature indicates that confronting weight status may negatively impact treatment engagement and outcomes; thus, the lack of focus on these issues in this study highlights a strength [89,90]. Delivery of RDMI in this study instead focused on diet quality, which can encompass a variety of factors (e.g., added sugars, fruits/vegetables). While such dietary factors may subsequently influence weight status, focusing on a flexible change goal enables greater individual autonomy, allowing weight-related discussions per participant preference, but not forcing them. This finding is further augmented by the use of RDs, who may be better equipped to navigate such conversations [91].

Despite MI’s role in addressing ambivalence [92], few studies measure ambivalence as either an outcome or potential mediator/moderator. This includes studies employing “opportunistic MI”, that is, MI for individuals who did not specifically present to receive MI/counseling [92,93]. Such approaches are promising, though often short in duration [94]. This study was opportunistic in the mechanism by which MI was presented to individuals, but maintained a more traditional length/duration. Interestingly, we found lower ambivalence made PACs less likely to be *RDMI Completers*, while baseline Change scores were negatively correlated with RDMI use. That is, those with higher ambivalence used more RDMI, and sustained a decrease in this ambivalence. Though these results cannot be attributed to RDMI alone, they do suggest that increasing RDMI’s reach to those with high ambivalence may improve engagement in interventions and outcomes.

This study had numerous strengths, including a detailed examination of MI from a RD, including in a diverse cohort, as well as for the purposes of modifying PAC behavior in the context of a child-focused pediatric obesity prevention intervention. However, this study is not without limitations. As data for one year of the overarching five-year trial are presented, the sample size is small, limiting interpretation and potentially explaining the absence of statistical significance. In addition, in this study, the short-term follow up may be insufficient to observe sustained changes in health or behavior, and limit the interpretation of long-term or sustained implications. In conclusion, results indicate MI from a RD delivered to PACs can improve ambivalence regarding diet quality, as well as measures of diet, in both PACs and youth, including among PACs with high ambivalence.

## 5. Conclusions

This research provides preliminary support for the use of MI from a RD to directly target PAC diet and indirectly target child diet and the home environment as a means of obesity prevention, and to improve cancer prevention and control efforts. Findings further indicate that this approach led to favorable results in PACs with higher baseline ambivalence, while contributing to decreases in ambivalence, consistent with the theoretical mechanisms of MI. Such intervention strategies are critical to simultaneously target adult and youth behavior as a key means of obesity and cancer prevention and control.

## Figures and Tables

**Table 1:** Demographic and Health Profiles of Parents and Adult Caregivers receiving Motivational Interviewing from a Registered Dietitian (RDMI).

Variable	Levels	RDMI Completers (n = 16) % (n)	SHA Completers (n = 24) % (n)	Entire Cohort (n = 36) % (n)
Age (years, mean (SD))	*-*	38 (6)	37 (5)	36 (5)
Sex	Female	81 (13)	83 (20)	89 (32)
	Male	19 (3)	17 (4)	11 (4)
Race	Black/African American	44 (7)	46 (11)	50 (18)
	White	44 (7)	42 (10)	36 (13)
	Mixed Race	6 (1)	8 (2)	8 (3)
	Prefer not to answer	6 (1)	4 (1)	6 (2)
Marital Status	Married	44 (7)	42 (10)	39 (14)
	Never Married	19 (3)	29 (7)	25 (9)
	Member of an Unmarried Couple	13 (2)	13 (3)	19 (7)
	Divorced	19 (3)	13 (3)	11 (4)
	Separated	6 (1)	4 (1)	6 (2)
Education Level	Grade 12 or Higher	56 (9)	4 (1)	6 (2)
	1–3 Years of College	44 (7)	50 (12)	50 (18)
	4 Years or More of College	0 (0)	46 (11)	44 (16)
Employment Status	Employed or Self-Employed	94 (15)	92 (22)	83 (30)
	Unemployed	6 (1)	4 (1)	8 (3)
	Unable to Work	0 (0)	4 (1)	6 (2)
	Student	0 (0)	0 (0)	3 (1)
BMI (kg/m^2^, mean (SD))	*-*	31.1 (6.11)	30.4 (6.04)	30.3 (5.67)
HEI Scores ^a^	*-*	61.5 (9.93)	62.3 (9.98)	62.9 (9.6)
Change Scores (i.e., ambivalence) ^b^	*-*	8.8 (1.30)	9.1 (1.13)	9.0 (1.1)

^a^ HEI-2015 scores indicate diet quality as determined by adherence to the 2015–2020 US Dietary Guidelines for Americans, scores range from 0–100, with higher scores indicating higher diet quality; ^b^ Change Scores obtained from the Change Questionnaire, scores range from 0–10, with higher scores indicating lower ambivalence. RDMI = Motivational Interviewing from a Registered Dietitian; SHA = Summer Harvest Adventure; SD = standard deviation; BMI = body mass index; HEI = Healthy Eating Index.

**Table 2:** Multiple Linear Regression for Measures of RDMI Use, Summer Harvest Adventure Attendance, and Changes in PAC Diet Quality ^a^.

Variables (n = 24) ^b^	B ^c^	SE B	Β ^d^	*p*	95% CI
RI Dose Received (Total Model Adjusted R^2^ = 0.002; f^2^ = 0.096)					
RI Dose Received	0.571	0.894	0.145	0.530	−1.289, 2.431
SHA Attendance	1.986	2.184	0.207	0.374	−2.556, 6.528
RDMI Dose Received (Total Model Adjusted R^2^ = 0.055; f^2^ = 0.159)					
RDMI Dose Received	0.980	0.773	0.271	0.219	−0.629, 2.588
SHA Attendance	1.708	2.054	0.178	0.415	−2.564, 5.979
RDMI Time Received (Total Model Adjusted R^2^ = 0.089; f^2^ = 0.202)					
RDMI Time Received	0.055	0.035	0.344	0.131	−0.018, 0.129
SHA Attendance	1.169	2.101	0.122	0.584	−3.199, 5.537
RDMI Engagement (Total Model Adjusted R^2^ = 0.095; f^2^ = 0.211)					
RDMI Engagement	0.071	0.044	0.328	0.121	−0.020, 0.163
SHA Attendance	1.877	1.947	0.196	0.346	−2.171, 5.925

^a^ Diet quality assessed using Healthy Eating Index 2015 scores; ^b^ PACs who completed baseline and post-intervention assessments included in analyses; ^c^ unstandardized coefficient; ^d^ standardized coefficient. RDMI = Motivational Interviewing from a Registered Dietitian; SHA = Summer Harvest Adventure; PAC = parent/adult caregiver; SE = standard error; RI = reciprocal interactions.

**Table 3:** Multiple Linear Regression for Measures of RDMI Use, Summer Harvest Adventure Attendance, and Changes in Child Skin Carotenoids.

Variables (n = 24) ^b^	B ^c^	SE B	Β ^d^	*p*	95% CI
RI Dose Received (Total Model Adjusted R^2^ = −0.055; f^2^ = 0.037)					
RI Dose Received	1.315	2.416	0.128	0.592	−3.710, 6.340
SHA Attendance	−5.096	5.901	−0.202	0.398	−17.368, 7.176
RDMI Dose Received (Total Model Adjusted R^2^ = −0.067; f^2^ = 0.027)					
RDMI Dose Received	0.591	2.158	0.062	0.787	−3.898, 5.079
SHA Attendance	−4.303	5.733	−0.171	0.461	−16.225, 7.619
RDMI Time Received (Total Model Adjusted R^2^ = −0.059; f^2^ = 0.034)					
RDMI Time Received	0.047	0.100	0.111	0.643	−0.161, 0.255
SHA Attendance	−4.966	5.952	−0.197	0.414	−17.345, 7.413
RDMI Engagement (Total Model Adjusted R^2^ = −0.068; f^2^ = 0.026)					
RDMI Engagement	−0.027	0.126	−0.047	0.823	−0.288, 0.234
SHA Attendance	−3.541	5.556	−0.141	0.531	−15.096, 8.014

^b^ unstandardized coefficient; ^c^ standardized coefficient; ^d^ standardized coefficient. RDMI = Motivational Interviewing from a Registered Dietitian; SHA = Summer Harvest Adventure; PAC = parent/adult caregiver; SE = standard error; RI = reciprocal interactions.

**Table 4:** Multiple Linear Regression for Measures of RDMI Dose, Summer Harvest Adventure Attendance, and Changes in PAC Modeling ^a^.

Variables (n = 24) ^b^	B ^c^	SE B	Β ^d^	*p*	95% CI
RI Dose Received (Total Model Adjusted R^2^ = 0.144; f^2^ = 0.279)					
RI Dose Received	3.559	1.484	0.506	0.026 *	0.471, 6.646
SHA Attendance	−2.418	3.625	−0.141	0.512	−9.957, 5.121
RDMI Dose Received (Total Model Adjusted R^2^ = 0.036; f^2^ = 0.136)					
RDMI Dose Received	2.325	1.400	0.359	0.112	−0.586, 5.236
SHA Attendance	−0.898	3.717	−0.052	0.811	−8.628, 6.832
RDMI Time Received (Total Model Adjusted R^2^ = 0.016; f^2^ = 0.124)					
RDMI Time Received	0.099	0.066	0.344	0.146	−0.038, 0.236
SHA Attendance	−1.371	3.913	−0.080	0.730	−9.508, 6.767
RDMI Engagement (Total Model Adjusted R^2^ = −0.037; f^2^ = 0.057)					
RDMI Engagement	0.088	0.084	0.228	0.307	−0.087, 0.264
SHA Attendance	0.265	3.733	0.015	0.944	−7.499, 8.029

^a^ Modeling measured using the Home Self-Administered Tool for Environmental Assessment of Activity and Diet (HomeSTEAD); ^b^ PACs who completed baseline and post-intervention assessments included in analyses; ^c^ unstandardized coefficient; ^d^ standardized coefficient. RDMI = Remote Motivational Interviewing from a Registered Dietitian; SHA = Summer Harvest Adventure; PAC = parent/adult caregiver; SE = standard error; RI = reciprocal interactions; * *p* ≤ 0.05.

## Data Availability

Data may be made available upon reasonable request.

## Supplementary Materials

The following supporting information can be downloaded at: https://www.mdpi.com/article/10.3390/ijerph20064726/s1, Table S1: RDMI Use Among Parents and Adult Caregivers; Table S2: Logistic Regression for Prediction of Classification as a RDMI Completer versus Non-Completer.
