# Supplementary material for: Preliminary Support for the Use of Motivational Interviewing to Improve Parent/Adult Caregiver Behavior for Obesity and Cancer Prevention
Source: Int J Environ Res Public Health. 2023 Mar 7;20(6):4726. doi: 10.3390/ijerph20064726 (PMC10048747; doi:10.3390/ijerph20064726)
Supplement: Supplementary file 1 [file ijerph-20-04726-s001.zip › Supplementary Table S1 RDMI Use Among Parents and Adult Caregivers.pdf]

Supplementary Table S1: RDMI Use Among Parents and Adult Caregivers

Variable	RDMI Completers (n=16) Mean (SD)	SHA Completers (n=24) Mean (SD)	Entire Cohort (n=36) Mean (SD)
RI Dose Received[a]	5.1 (1.18)	4.1 (1.74)	2.4 (1.96)
RDMI Dose Received[b]	4.1 (1.36)	3.0 (1.9)	3.3 (2.11)
RDMI Time Received[c] ***(minutes)***	66.3 (41.64)	47.6 (42.3)	37.9 (39.07)
RDMI Engagement[d] ***(%)***	70.0 (22.00)	50.0 (31.00)	41.0 (33.00)

RDMI provided to parents and adult caregivers enrolled in an obesity prevention intervention and randomized to intervention (SHA) versus control (Midwest, US)

[a]*RI Dose Received* includes any interaction from the RD to which the participant responded in any capacity;

[b]*RDMI Dose Received* includes any telephonic interaction that did not end in the participant stating they did not want to talk or had to reschedule (i.e., full RDMI was provided);

[c]*RDMI Time Received* computed as the sum of all time of RDMI doses received, in minutes;

[d]*RDMI Engagement* quantified as number of RDMI doses received divided by total number of attempted RDMI interactions.
