# Supplementary material for: Preliminary Support for the Use of Motivational Interviewing to Improve Parent/Adult Caregiver Behavior for Obesity and Cancer Prevention
Source: Int J Environ Res Public Health. 2023 Mar 7;20(6):4726. doi: 10.3390/ijerph20064726 (PMC10048747; doi:10.3390/ijerph20064726)
Supplement: Supplementary file 1 [file ijerph-20-04726-s001.zip › Supplementary Table S2 Logistic Regression for Prediction of Classification.pdf]

Supplementary Table S2: Logistic Regression for Prediction of Classification as a RDMI Completer versus Non-Completer

Variable (n=36)[a]	B[b]	SE	P	OR	95% CI
Baseline PAC BMI	0.077	0.073	0.293	1.080	0.936, 1.245
Baseline child BMI percentile	-0.003	0.015	0.839	0.997	0.969, 1.026
Baseline PAC HEI score	-0.008	0.044	0.862	0.992	0.910, 1.082
Baseline Change score	-0.428	0.385	0.266	0.652	0.307, 1.386
African American	-0.606	0.849	0.475	0.545	0.103, 2.879

[a]All PACs randomized to the Summer Harvest Adventure included in analysis; [b]Unstandardized coefficient.

SE=standard error; OR=odds ratio; PAC=Parent/Adult Caregiver; BMI=body mass index; HEI=Healthy Eating Index 2015; *RDMI Completer* status assigned based on continued RDMI phone calls throughout the intervention, or reaching a collaborative agreement that calls were no longer needed
